# Cost-Effective and Efficient Cool Nanopigments Based on Oleic-Acid-Surface-Modified ZnO Nanostructured

**DOI:** 10.3390/ma16062159

**Published:** 2023-03-08

**Authors:** Ashraf H. Farha, Abdullah F. Al Naim, Shehab A. Mansour

**Affiliations:** 1 Department of Physics, College of Science, King Faisal University, Al Ahsa 31982, Saudi Arabia; anaim2@kfu.edu.sa; 2 Department of Physics, Faculty of Science, Ain Shams University, Cairo 11566, Egypt; 3 Basic Engineering Science Department, Faculty of Engineering, Menoufia University, Shebin El-Kom 32551, Egypt; shehab_mansour@yahoo.com; 4 Advanced Materials/Solar Energy and Environmental Sustainability (AMSEES) Laboratory, Faculty of Engineering, Menoufia University, Shebin El-Kom 32551, Egypt

**Keywords:** nanopigments, ZnO nanocrystals, NIR reflectance, thermal decomposition, surface modification, oleic acid, cool-nanopigment applications

## Abstract

In this paper, as-synthesized and oleic acid (OA)-surface-treated zinc oxide (ZnO) nanocrystals were successfully synthesized and investigated for cool-nanopigment applications. ZnO nanocrystals were synthesized using the thermal decomposition method. The OA-surface-treated ZnO sample was obtained with an OA:ZnO ratio of 1:1. The structural, optical and morphological properties of the samples were characterized via X-ray diffraction (XRD), Fourier transform infrared (FTIR), UV-VIS-NIR diffused reflectance spectroscopy (DRS) and field-emission scanning electron microscopy (FE-SEM) techniques. ZnO nanocrystals possess a well-known zincate phase of ZnO, as confirmed for the as-synthesized sample with a reduction in the integrity of the ZnO crystalline structure upon the application of the OA coating. XRD peaks broadening and decreasing in crystallite size were obtained upon the surface modification of the ZnO by OA. The average crystallite size decreased from 31.5 to 17.4 nm, and the surface area changed from 23.37 to 7.48 m^2^/g upon OA treatment. These changes were attributed to the well-capping of the ZnO nanoparticles by OA, and, furthermore, OA increased the dispersion of the nanoparticles. The optical band gap of the investigated samples demonstrated a blue shift from 3.06 eV to 3.22 eV upon treatment. Both samples showed high NIR reflectance ($R_{NIR}^{*}$) values, which makes them well qualified for use as cool nanopigments. Additionally, the as-synthesized sample showed an $R_{NIR}^{*}$ value higher than that of the treated sample.

## 1. Introduction

In recent decades, materials with nanoscale particle sizes have been of great interest due to their high surface area, one of their novel properties making them applicable in many fields. Among such nanoscale materials is zinc oxide (ZnO) ceramic of II–VI oxides. Generally, ZnO and ZnO-based compounds have received much attention due to their extensive applications [1,2]. Evidently, these extensive applications of ZnO are the result of its valuable properties. The most important properties that ZnO possesses include its direct band gap at 3.3 eV, excellent chemical and thermal stabilities, low cost, nontoxic nature and premium photo- and piezoelectric effects. An important aspect of the direct band gap of ZnO is the formation of bound excitons, which are related electronic states within the band gap. Such states are due to defects, which alter the structure of the band and, hence, change the optical absorptions and emissions of the material [3]. In addition to the well-known applications of ZnO in LCD displays, gas sensing and windows in solar cells [1,4,5], it has also been extensively used for drug delivery in biomedical applications [6,7]. Recently, interest has grown in using ZnO in cool-painting applications [8,9,10]. Such types of applications require materials with a high NIR reflection, such as ZnO, in order to use them in cool painting for exterior surfaces. Such paintings have an excellent impact on the reduction in the energy required for cooling [2].

In addition to all the above good properties of ZnO, it also has some drawbacks that affect its performance in optoelectronic devices. These limitations include its high resistivity, its low transparency in the UV region, its low concentration of charge carriers and its tendency to form high agglomerations [1,2,3,11]. Therefore, various studies have been carried out to overcome these limitations without impacting the performance properties of ZnO.

The doping of ZnO using one or more metal elements of group III has been employed to enhance both its electrical conductivity and optical transparency [11,12,13,14]. In our previous works on ZnO, the polymer pyrolysis method was used with three different types of initiators of the polymerization, namely, ammonium persulfate (APS), sonochemical (SON) and γ-irradiation, in order to form well-dispersed zinc oxide nanocrystals [2,15], giving lots of advantages to this method [2,16]. The properties of nanoparticles provide nanopigments with many advantages over conventional pigments. The high surface area allows for more resin adsorption on the surface and, hence, a larger coating fraction. Moreover, the colors of paints with nanopigments can change due to an alternating electrical field [17]. Using nanopigments increases hardness and resistance to scratches [17,18]. Several synthesis methods have been used to obtain nanopigments, such as the sol–gel method [2], the hydrothermal sonochemical hydrolysis technique [19], γ-radiation-assisted polymer pyrolysis methods [2] and thermal decomposition [20].

Another method that can be used to obtain highly dispersed ZnO nanoparticles (NPs) is the surface modification of ZnO. The functionalization of ZnO NPs with surfactants changes their properties, increasing the range of their applications. Improvements in the thermal stability and permeability of polysulfone membranes for ZnO NPs functionalized with oleic acid have been reported [21]. The surface functionalization of ZnO has been carried out using many organic and inorganic media (c. f. [1]). One of the approaches that has been applied to enhance the surface modification of zinc oxide is the use of organic compounds as surfactants to affect the formation of ZnO particles. Nanopigments have been recently applied in a wide range of applications, such as cosmetics, antimicrobial paints, automobile coatings, self-cleaning coatings, anti-UV coatings, self-healing coatings and ceramic decoration [2,7,18,20,22].

Zhao et al. [23] stated that the decomposition of zinc acetate begins at 210–250 °C and that complete decomposition into ZnO and other organic compounds occurs at 400 °C, and they also obtained ZnO NPs with a size of 20–30 nm.

In the present work, on the one hand, ZnO NPs were prepared by using the thermal decomposition method, which is an effective low-cost and high-yield method, after the calcination of zinc acetate salt, taking into account its low decomposition temperature [23]. Two of the greatest problems with nanopigments are their poor mixing and high agglomeration, which can be enhanced via surface functionalization. Then, on the other hand, the surfaces of the ZnO NPs were functionalized using oleic acid. This decreases the agglomeration rate, improves the optical properties and crystallinity, and controls the particle size. Functionalized OA forms a compact covalent bond between ZnO nanoparticles and the organic matrix of oleic acid through the creation of hydroxyl groups on their surfaces [24]. These effects of OA surface treatment on the structural, morphological, optical and cool NIR solar reflectance ($R_{NIR}^{*}$) calculations for the nanopigment application features of ZnO NPs were studied. It was expected that the surface functionalization of ZnO NPs by OA would enhance their compatibility and dispersibility within the resins of painting applications.

## 2. Experimental Details

### 2.1. Synthesization of ZnO and Oleic-Acid-Treated ZnO Nanocrystals

**Figure 1:**
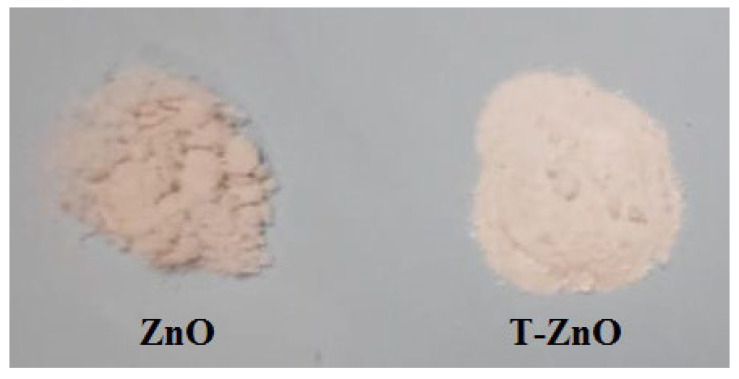
Photograph image for ZnO and T-ZnO nanopigment powders.

An amount of 15 g of zinc acetate dihydrate (Winlab, purity: 99.999%) in an alumina crucible were exposed to continuous heating in air at 350 °C for 2.5 h. The typical preparation process of the thermal decomposition technique was reported elsewhere [20,25]. The procedure of the surface treatment of ZnO NPs using oleic acid (OA) was reported elsewhere [26]. In the typical method, 1 g from ZnO NPs, 60 mL water and 3 mL OA were introduced into a glass beaker, and then the mixture was exposed to ultrasonication for 30 min. Thereafter, the ZnO-OA mixture was stirred for 8 h using the magnetic stirrer for completion of the reaction. After the reaction was completed, the obtained mixture was centrifuged for 1 h. Then, 60 mL of ethanol were used to wash the obtained precipitate. After that, the precipitate was set in ultrasonication for 30 min and centrifuged for 1 h. The washing, ultrasonication and centrifuging steps were repeated three times. Then, the final product of OA-ZnO was attained by drying the obtained precipitate at room temperature (RT) under vacuum conditions. The obtained as-synthesized powder sample is named ZnO sample. However, the OA-surface-treated ZnO sample is named T-ZnO sample. The obtained color for both investigated samples seems to be off-white, as commonly obtained for ZnO. The photographs of ZnO and T-ZnO powder are shown in Figure 1. The obtained photographs refer to the closeness in color for both samples. However, the T-ZnO has more brightness than the ZnO sample. More details about the color description of the investigated samples will be discussed later in Section 3.5 Color Coordinate Characterization.

### 2.2. Characterizations

The structures of the as-synthesized and OA-surface-treated ZnO were achieved from X-ray diffraction (XRD) technique and Fourier transform infrared (FTIR) measurements. A Philip’s X-ray diffractometer (MPD 3040) equipped with CuKα X-ray source of wavelength λ = 1.5406 Å was employed for XRD measurements. The X-ray diffraction measurements were run over a diffraction angle, 2θ ranging from 10 to 80° in 0.02° angle-step. FTIR transmittance spectra of the samples were acquired by using a JASCO (FT/IR-4100) spectrometer over wave numbers ranging from 400 to 4000 cm^−1^. The morphological and surface features of the samples were checked using the field-emission scanning electron microscope (FE-SEM), type Quanta FEJ20. A UV/Vis/NIR V570- JASCO spectrophotometer was used for the diffused reflectance measurements over an incident light wavelength ranging from 200 to 2500 nm.

Brunauer–Emmett–Teller (BET) surface area measurements of the samples were carried out using a Micromeritics Gemini 2375 nitrogen adsorption analyzer. The samples were exposed to degassing at 80 °C for 2 h under nitrogen gas flow prior to the surface area measurements.

The colorimeter FRU model WR-10 was used for measuring the color coordinates of the investigated nanopigments. The measurements were performed according to CIE recommended 1976 L* a* b* colorimetric mode. Given that L* is the color lightness coordinate (L* = 0 for black and L* = 100 for white), the coordinates a* and b* vary from green and blue for negative values to red and yellow for positive values, respectively.

## 3. Results and Discussion

### 3.1. Structural Characterization of ZnO and Oleic Acid-Treated ZnO Nanocrystals

**Figure 2:**
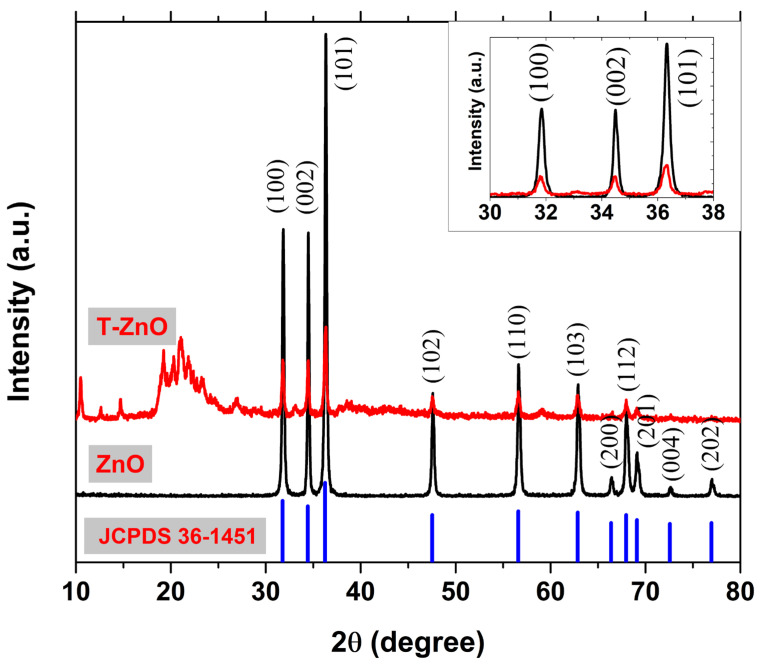
XRD patterns of ZnO and OA-treated ZnO samples with ZnO JCPDS standard card data. For more clarification, an enlargement of the ZnO highest three peaks; (100), (002) and (101) is demonstrated in the inset.

XRD results of both ZnO and T-ZnO samples are depicted in Figure 2. The XRD peaks of the pristine ZnO sample show good agreement with that of the standard data of wurtzite hexagonal structure. The absence of any other peaks is confirmed by obtaining a wurtzite structure long-range order ZnO sample with no other peaks that could be related to any other phases. Both samples showed a high texture in (101) orientation as the (101) peaks showed the highest intensity compared to all the other peaks. The inset of Figure 2 shows an enlargement for the three most intense XRD peaks with the orientations, namely (100), (002) and (101) for both samples. There are clear decreases in the intensity of these XRD peaks of the T-ZnO sample compared to those of the as-synthesized one, as shown in inset of Figure 2. The decrease in the XRD peak’s intensity is an indication of lowering the degree of crystallinity of the T-ZnO sample compared to the as-synthesized one. The degree of crystallinity of the samples was calculated from XRD results. The degree of crystallinity of the T-ZnO sample is 13.45% and that of the as-synthesized ZnO sample is 83.75%. The average crystallites size, D, at Bragg diffraction angle, θ was calculated using a Scherer equation, $D=\frac{0.94\lambda }{B_{hkl}\cos\theta }$, given λ = 1.5406 Å, and $B_{hkl}$ is the FWHM of the selected XRD peaks. The *D* values were found to be 33.1 and 16.1 nm for ZnO and T-ZnO samples, respectively. Additionally, *D* values were calculated according the Williamson–Hall equation, $B_{hkl}\cos\theta =\frac{0.94\lambda }{D}+4\varepsilon \sin\theta$, where ε is the induced strain in the samples [27]. The Williamson–Hall (WH) method gives the average crystallites sizes of 31.5 and 17.4 nm, for the ZnO and T-ZnO samples, respectively. From the XRD average crystallite size results one be able to notice that T-ZnO shows peaks broadening when compared to that of the pristine ZnO of much more higher and narrower peaks. Therefore, the surface modifications using OA after the synthetization of ZnO NPs would help in the size reduction of ZnO NPs in agreement with that reported by [24,28,29].

The lattice constants of the samples are calculated from the XRD results according to the ZnO wurtzite structure. The wurtzite ZnO lattice constants *a* and *c* showed very little variations for both samples. The average crystallites size (*D*), the lattice constants *a* and *c*, *c/a*, and the unit cell volume (*V*) of the ZnO and T-ZnO NPs were tabularized in Table 1.

**Figure 3:**
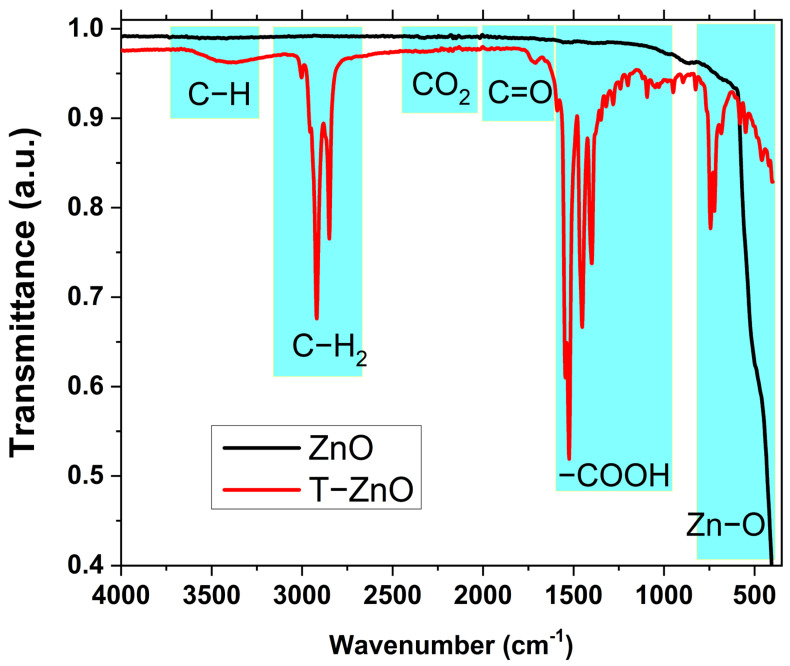
The transmittance FTIR spectra of ZnO and T-ZnO NPs samples.

FTIR investigation was used for a profound check on the effect of the OA surface treating on the structure of ZnO. Figure 3 shows the FTIR spectra of both pristine and OA-treated ZnO NPs. There are important absorption bands visible in the FTIR spectra of both ZnO and T-ZnO NPs. Starting from the lower wave number, a broad band range (400–700 cm^−1^) is visible with the highest intensity for untreated ZnO, and was shifted to a higher wave number for the OA-surface-treated sample. This band is due to the ZnO stretching mode vibration of ZnO for Zn and O bonds [30]. Additionally, the as-synthesized ZnO showed very weak absorption bands at 857 cm^−1^ which is related to Zn-O vibration. There are other very weak bands at 1536, 1654, for untreated ZnO NPs, which are due to both asymmetric and symmetric C=O stretching bonding. The absorption band at 2338 cm^−1^ and the other wide band at 3417 cm^−1^ correspond, respectively, to the adsorption of CO_2_ from the atmosphere and O-H vibration mode from zinc acetate [16]. These two bands are showing stronger absorption peaks in the T-ZnO sample than the pristine ZnO sample as a result of capping of ZnO NPs by the oleic acid molecules [24]. Such results prove that OA treatment results in in extremely well-dispersed ZnO NPs [30].

There are some strong absorption bands that appeared only in the T-ZnO spectrum, which are considered characteristic peaks of free oleic acid. These peaks include asymmetric and symmetric stretching modes of −CH_2_ at 2851 and 2920 cm^−1^, respectively. In addition, C=O stretching vibration bonding in OA appears at 1701 cm^−1^. The very strong bands at 1396–1456 cm^−1^ and 1526 cm^−1^ are due to symmetric and asymmetric carboxylate stretching of oleate. The carboxylates are chemisorbed onto the surface of ZnO NPs by covalent bonding [30]. The formation of all such OA-related peaks confirm the achievement of surface functionalization of ZnO NPs.

### 3.2. BET Surface Area Measurements of the Synthesized ZnO and T-ZnO Nanoparticles

Table 2 shows BET surface area results, pore radius and total pore volume of ZnO and T-ZnO samples. The BET surface area of ZnO NPs shows a decrease as the surface of the ZnO nanoparticle is treated with OA. Such a decrease in surface area from 23.37 to 7.48 m^2^/g on OA treating could be attributed to the well-capping of the ZnO NPs by OA, which will be discussed in the results of the FE-SEM micrographs. Moreover, the T-ZnO has lower values of average pore radius and total pore volume in comparison with the as-synthesized ZnO sample, which refers to the homogenous treatment as well as a good interaction between OA and ZnO NPs.

### 3.3. Morphological Characterization of the Synthesized ZnO and T-ZnO Nanoparticles

**Figure 4:**
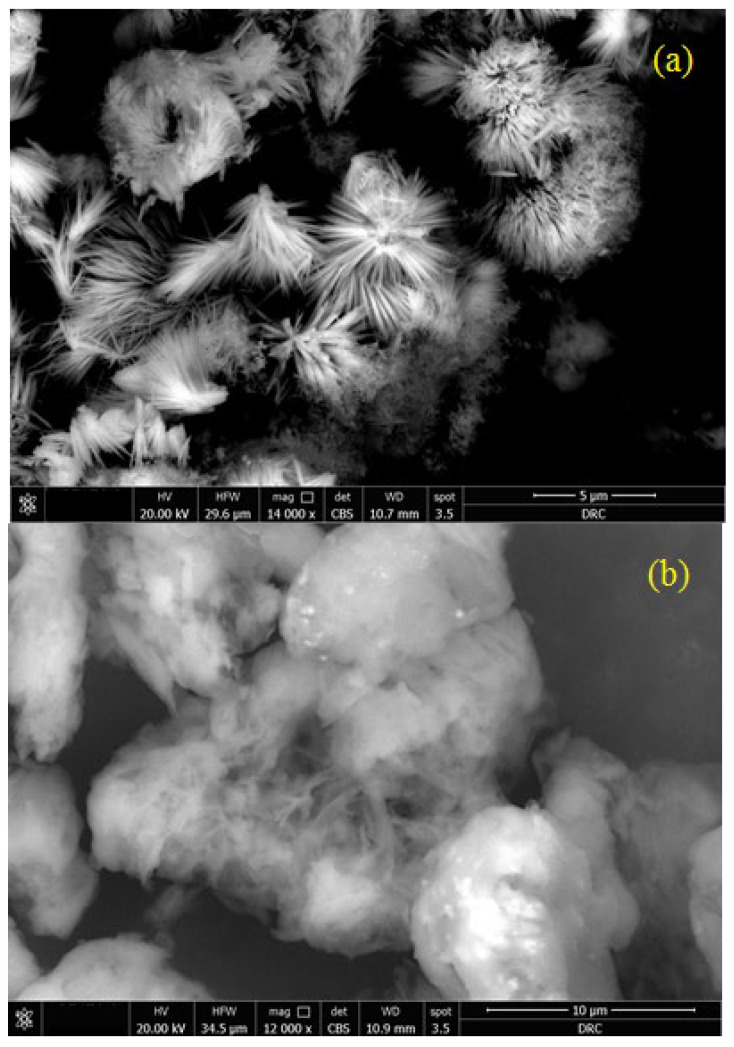
FE-SEM micrographs of: (**a**) ZnO NPs at 14,000× magnification, and (**b**) T-ZnO NPs at 12,000× magnification.

FE-SEM micrographs of ZnO and T-ZnO NPs are shown in Figure 4. The obtained shapes in the as-synthesized ZnO sample are nanorod shapes with agglomeration zones in flower-like features as illustrated in Figure 4a. The obtained flower-like ZnO nanorods enable larger surface areas which result in a good interaction of ZnO NPs with the OA treatments. The formation of a one-dimensional (1D) nanostructure shape, nanorods, in the as-synthesized ZnO is commonly reported for the thermal decomposition route of ZnO samples, see for example [25]. Figure 4b shows the micrograph of T-ZnO NPs. Such micrograph refers to a successful capping of ZnO nanorods by OA. However, the whole particles appear micron-sized, which indicate the significant effect of surface treatment by OA on the nanoparticle morphologies.

### 3.4. Optical Characterization of the Synthesized ZnO and T-ZnO Nanopigments

**Figure 5:**
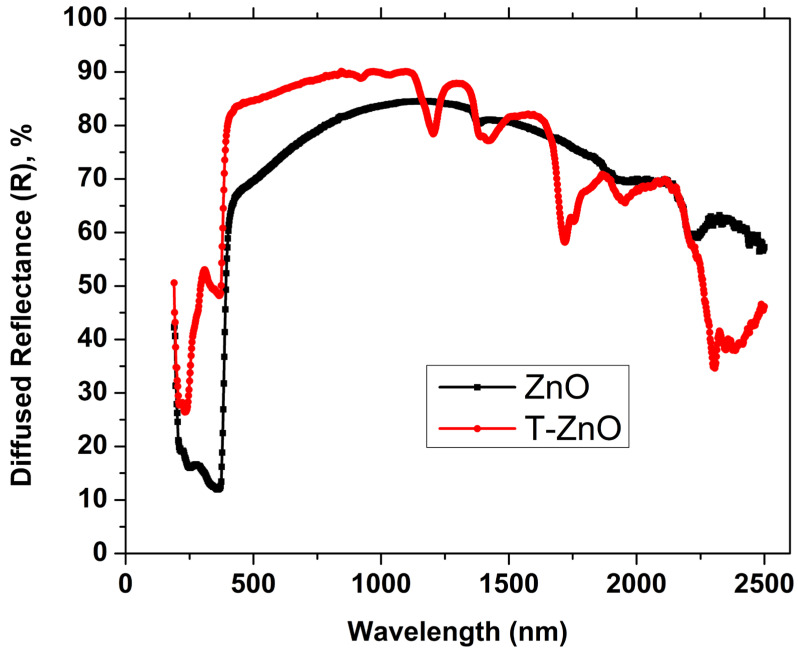
UV/Vis/NIR diffuse reflectance spectra (DRS) of ZnO and T-ZnO nanopigment samples.

Diffused reflectance spectroscopy (DRS) spectra of the ZnO and T-ZnO nanopigments are shown in Figure 5. The DRS spectra exhibited strong absorption bands at 370 nm and 368 nm for the ZnO and T-ZnO nanopigments, respectively. Such strong absorption bands are assigned to optical transitions of the optical energy band gap [31]. For the T-ZnO sample, the reflectance (*R*) reached maximum value (84.5%) at 1132 nm, which is higher than the maximum value of the pristine ZnO that was prepared by a similar technique but with a higher decomposition time, as reported in our previous work [20]. Indeed, the decomposition time and decomposition temperature have significant effects on the density of zinc interstitials and oxygen vacancies in ZnO nanocrystals [32,33]. The DSR spectrum of the pristine ZnO in the previous study [20], showed lower reflectance values than that obtained for the present investigated ZnO sample throughout the whole studied range of wavelengths. This result confirms that using a low decomposition time, as in the case of the present study, enables a lower density of lattice defects to be obtained, and therefore the absorbance is limited compared to the longer time of decomposition. For the T-ZnO sample, the maximum value of R is 90.1% and was recorded at a wavelength of 1096 nm. Moreover, from the low-wavelength range up to 1170 nm, there are significant increases in R values for T-ZnO in comparison to those obtained for the ZnO sample. Additionally, the same result is found in the high wavelength range after 1170 nm. Such high values of R for the T-ZnO sample could be present in this sample as an efficient white cool nanopigment. Calculated values of NIR solar reflectance ($R_{NIR}^{*}$) give an accurate illustration of the figure of merit of the investigated samples to be employed as cool nanopigments. Here, it is significant noting that NIR radiation, which is the greatest source of heating, represents approximately 52% of the solar radiation [34]. So, $R_{NIR}^{*}$ values in this region (700–2500 nm) were calculated according to the ASTM (G173-03) using the following equation [35]:

(1) $$R_{NIR}^{*}=\frac{\int_{700}^{2500}R\left(\lambda \right)i\left(\lambda \right)d\lambda }{\int_{700}^{2500}i\left(\lambda \right)d\lambda }$$

where $R\left(\lambda \right)$ is the obtained reflectance from DRS spectra in Wm^−2^ and $i\left(\lambda \right)$ is the ASTM standard solar spectral irradiance measured in Wm^−2^ nm^−1^. The obtained values of $R_{NIR}^{*}$ were found to be 80.47% and 84.75% for ZnO and T-ZnO nanopigment samples, respectively. The increase in $R_{NIR}^{*}$ value by treating the ZnO sample with OA could be referred to the benefits of capping of ZnO NPs using chemical ligands, such as OA. Specifically, the surface modification of the nanoparticles using a chemical ligand enables a good separation of the nanoparticles, and that leads to an increase in the NIR reflectance as a result of the increase in density of the scattering sites. In addition to the enhancement in $R_{NIR}^{*}$ in the ZnO sample due to surface treating with OA, the obtained $R_{NIR}^{*}$ value for pristine ZnO NPs, 80.47%, is much higher than their corresponding values for the ZnO NPs that were prepared by other different methods, see for example [20,36]. A comparison of the $R_{NIR}^{*}$ values for the investigated nanopigments with other white nanopigments based on ZnO or TiO_2_ are tabulated in Table 3. Here, it worth mentioning that the obtained diffused reflectance values of ZnO prepared using the arc discharge technique have been reported between 14% and 54% in the NIR range (700–2500 nm) [36]. However, the $R_{NIR}^{*}$ value was found to be 64.8% for pure ZnO prepared by the same used technique of the investigated samples but with longer decomposition time [20]. On the other hand, the used preparation method in the current study is offering a facile and cost-effective method with reasonable values of $R_{NIR}^{*}$, 80.47% and 84.75%, in comparison with more complicated chemical methods with higher calcination temperatures, such as modified polymer pyrolysis with γ-irradiation, which registered ~88% for pure ZnO [2]. The superior characteristics of the investigated nanopigments are comparable to many of the other white nanopigments, due to their high efficiency in reflectivity and cost effectiveness.

**Figure 6:**
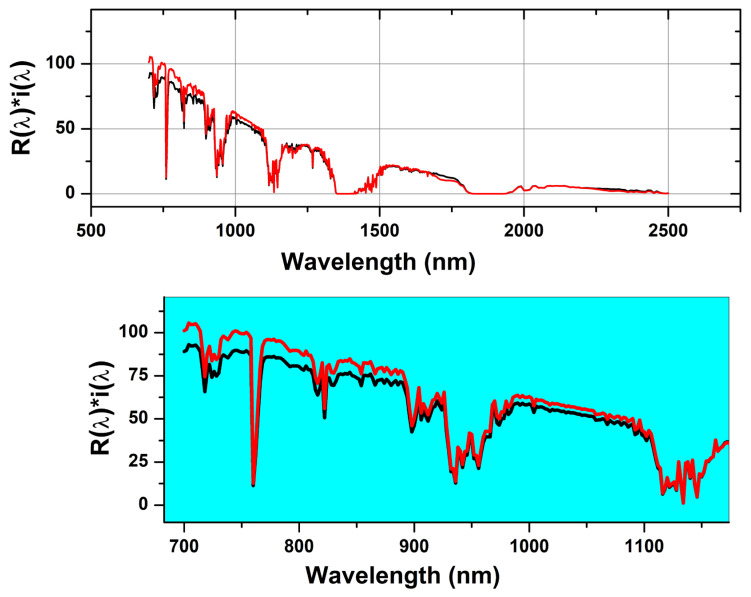
The effective reflectance, $R\left(\lambda \right)*i\left(\lambda \right)$ spectrum for ZnO (black color) and T-ZnO (red color) nanopigment samples using a real incident solar radiation according to the ASTM (G173-03).

The effective reflectance spectra, $R\left(\lambda \right)*i\left(\lambda \right)$, throughout the investigated NIR range, are shown in Figure 6 for ZnO and T-ZnO nanopigment samples. The spectra of both samples are very close in intensity and shape. However, there is quite a variation in the intensity of the wavelength range 700–1100 nm as shown in the zoomed part of Figure 6. In such a wavelength range, the effective reflectance intensity of T-ZnO is higher than that of the ZnO sample. This result could be incorrigible to the role of surface modification of nanoparticles in that causes decreasing of traps the density due to defects [38,39].

**Figure 7:**
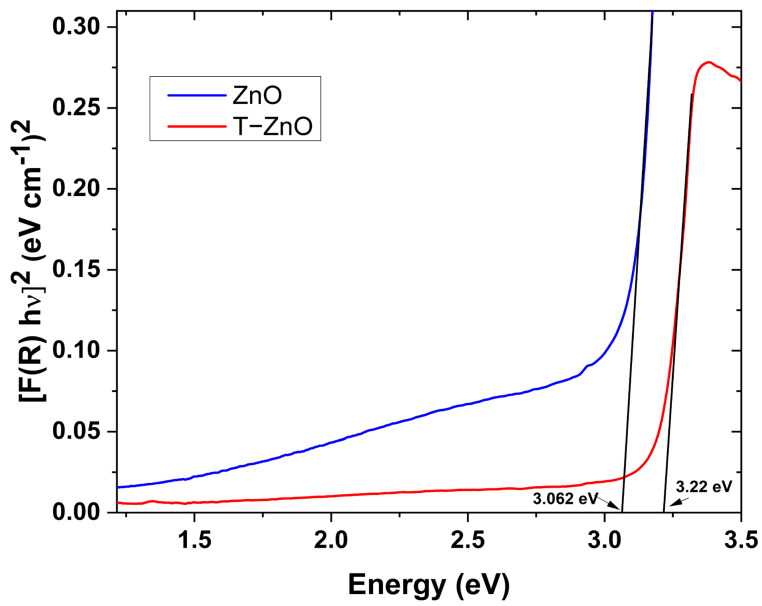
The variation of Kubelka–Munk function [*F(R)hν*]^2^ as a function of incident photon energy for ZnO and T-ZnO nanopigments.

The optical energy band gap (*E_g_*) of the investigated nanopigments were calculated uing the Kubelka–Munk (K-M) approach for powdered materials. Consequently, the function *F*(*R*)*,* K-M function, was estimated according the following equation [40]:

(2) $$F\left(R\right)=\frac{{\left(1-R\right)}^{2}}{2R}=\frac{k}{s}$$

where *R* is the obtained reflectance from DRS data. Nevertheless, the coefficients *k* and *s* are K-M absorption and scattering coefficients, respectively. The linear absorption coefficient (α) and the photon energy (hν) relation is usually used to determine *E_g_*, which is given for direct band gap semiconductors according to the subsequent equation [31]:

(3) $${\left(\alpha h\nu \right)}^{2}=C_{1}\left(h\nu -E_{g}\right)$$

where $C_{1}$ is a proportionality constant. In the K-M approach, *F(R)* is similar to the linear absorption coefficient (α) [41]. Specifically, *k* is considered equivalent to double the α value at 60° as an angle of incident. Moreover, the independency of *s* on the value of the wavelength led to a modification of Equation (3) to the following [31,41]:

(4) $${\left[F\left(R\right)h\nu \right]}^{2}=C_{2}\left(h\nu -E_{g}\right),$$

where the proportionality constant $C_{2}$ is constantly correlated to s and $C_{1}$. The previous equation was used to determine the *E_g_* value for both studied samples. In this respect, plots of ${\left[F\left(R\right)h\nu \right]}^{2}$ versus hν for ZnO and T-ZnO are shown in Figure 7. *E_g_* values that were obtained from the extrapolation of the linear part at the higher photon energy at ${\left[F\left(R\right)h\nu \right]}^{2}=0$ are 3.062 and 3.22 eV for ZnO and T-ZnO, respectively. Indeed, the formation of surface ligands on NPs has a significant effect on the energy level shift in NPs as a result of the formed moments, due to the nanoparticle/ligand interface dipole and the intrinsic dipole of the ligand molecule itself [42]. The probable shift in the energy level of NPs offers a good opportunity to increase the value of the optical band gap in comparison with the non-treated sample. It should be noted that the obtained *E_g_* values for both NPs under investigation are still smaller than that of bulk ZnO (3.37 eV). Such a reduction in optical band energy could be correlated to the higher density of defects in intergranular regions [2,43,44].

**Figure 8:**
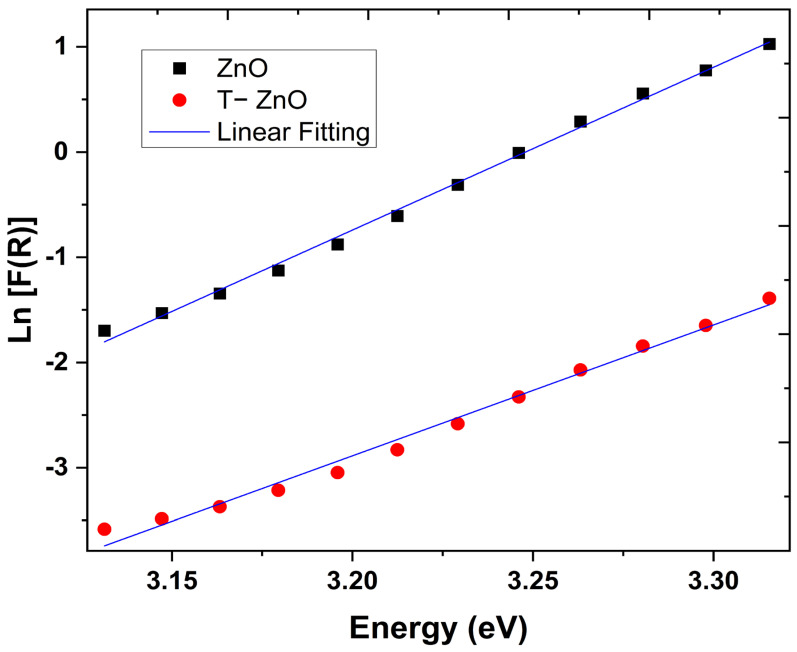
Plot of *ln* [*F(*R)] versus Energy (*hν*) for the investigated for ZnO and T-ZnO nanopigments.

One of the most important parameters to give more information about the disorder degree of the material is the Urbach energy (*E_U_*). The disorder in the basic structure of materials enables modifications of the bonding scheme of the host atoms/ions that emerged as extended states in the conduction, and valance bands are so-called the Urbach tail [45,46]. Accordingly, the *E_U_* values are calculated for the investigated samples to obtain information about the variation of disorder degree in the structure due to the introduction of OA. The linear absorption coefficient and the incident photon energy are correlated by the following equation [47]:

(5) $$\alpha =\alpha_{0}\;exp\left(h\nu /E_{U}\right),$$

where $\alpha_{0}$ is the material-dependent pre-exponential coefficient. Nevertheless, the optical absorption α, could be replaced by Kubelka−Munk function *F*(*R*) using the DRS data. Subsequently, Figure 8 shows the plots of *ln* [*F(R*)] vs. hν for the ZnO and T-ZnO samples. The curve for each sample gives a good straight line that was fitted using a linear least-square fitting. The inverse of the slope gives the *E_U_* value. The calculated *E_U_* values are 64.3 and 80.4 meV for ZnO and T-ZnO, respectively. This result indicates the higher disorder in the structure of the T-ZnO sample in comparison with the non-treated ZnO sample. The increase in the density of the tail states of ZnO NPs by surface treatment, using chemical ligand OA, confirms the good interaction between ZnO NPs and OA. Moreover, the increase in the disorder degree of T-ZnO supports their lower crystalline degree comparing to the non-treated sample as previously discussed in XRD data.

### 3.5. Color Coordinate Characterization

CIE L* a* b* describes the whole range of the colors which are visible to the human eyes and introduces a device-independent model to be used as a reference. The obtained values of (L*, a* and b*) were (84.34, 1.9 and 6.8) and (91.33, 0.95 and 7.09) for the ZnO and T-ZnO nanopigment samples, respectively. The T-ZnO sample exhibited a higher L* value than the ZnO sample, which indicates the increase in its lightness due to the surface treatment of NPs by OA. Indeed, CIE L* a* b* expresses the color space by two perpendicular axes a* and b* that are orthogonal to the vertical axis of lightness (L*). The color space in polar presentation, the hue is determined by the angle ($h^{o}$) and the Chroma or saturation (C*). Both $h^{o}$ and $C^{*}$ could be obtained using the following relations [48]:

(6) $$h^{o}=\tan^{-1}\left(\frac{b^{*}}{a^{*}}\right),$$

(7) $$C^{*}=\sqrt{{\left(a^{*}\right)}^{2}+{\left(b^{*}\right)}^{2}},$$

**Figure 9:**
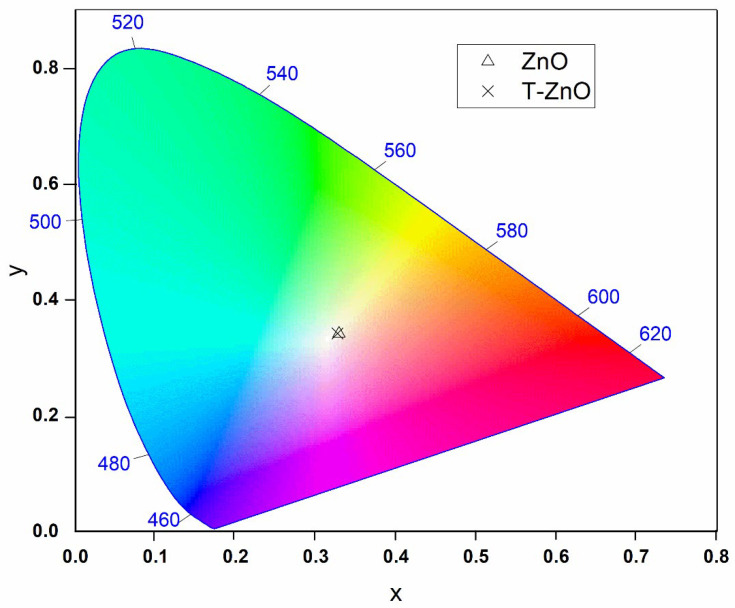
CIE 1931 color space chromaticity diagram in the (x, y) coordinates system for the investigated ZnO and T-ZnO nanopigments.

Using Equation (6), the hue angles of the investigated nanopigment samples were found to be 74.38 and 82.36° for the ZnO and T-ZnO nanopigment samples, respectively. The obtained values of $h^{o}$ of both samples indicate the tendency of the color to turn yellow with higher intensity for the T-ZnO sample. It worth mentioning that the higher intensity of yellow color is recorded at $h^{o}$ = 90° [48]. On the other hand, the obtained Chroma ($C^{*}$) values for both samples are closer to each other and were found to be 7.06 and 7.153 for ZnO and T-ZnO nanopigment samples, respectively. The color representation for the investigated nanopigments is shown in Figure 9 using the 1931 standard (CIE) color space chromaticity diagram in (x, y) coordinates. The obtained color for both nanopigment samples is a light yellow with close coordinates. The correlated color temperatures (CCT) for the studied nanopigments are 5657 and 5741 K for the ZnO and T-ZnO samples, respectively.

## 4. Conclusions

ZnO nanocrystals in powder form have been synthesized by a thermal decomposition method. The OA-surface-treated ZnO sample was obtained with an equivalent fraction of ZnO powder to OA. The XRD characterization of the as-synthesized sample confirmed a wurtzite structure. The surface modification of ZnO showed a significant effect on average crystallites sizes and crystallinity of the ZnO. A blue shift in the optical band gap upon surface treatment of ZnO nanocrystals was obtained. The value of Urbach tails at the edges of the valence and conduction bands showed an increase of 64.3 and 80.4 meV for ZnO and T-ZnO, respectively. The density of the tail states in ZnO increases upon OA surface treatment which confirms the well-capping interface of OA on ZnO NPs, as well as an enhancement of the dispersion of ZnO NPs being obtained. The preparation method in the current study grants a facile and cost-effective way to obtain reasonable values of $R_{NIR}^{*}$, (80.47% and 84.75%, for ZnO and T-ZnO, respectively) in comparison to other complicated chemical methods and/or high-temperature calcination conditions.

## Figures and Tables

**Table 1:** The crystallites size (*D*), the lattice constants (*c* and *a*), *c/a* and the cell volume (*V*) of the as-synthesized ZnO and OA-surface-treated ZnO NPs.

Sample	*D* (Scherer), nm	*D* (WH), nm	a, Å	c, Å	c/a	V, Å
ZnO	33.1	31.5	3.244	5.201	1.605	47.39
T-ZnO	16.1	17.4	3.248	5.206	1.604	47.56

**Table 2:** The surface area, average pore radius and total pore volume of ZnO and T-ZnO NPs.

Sample	Surface Area (m²/g)	Average Pore Radius (nm)	(TOPV) Total Pore Volume (cc/g)
ZnO	23.37	1.92	3.42 × 10^−2^
T-ZnO	7.48	1.63	1.48 × 10^−2^

**Table 3:** Comparison of $R_{NIR}^{*}$ values for the investigated nanopigments with those obtained for some various white nanopigments.

White Nanopigment	Synthesization Technique	$R_{NIR}^{*}$%	References
ZnO	Thermal decomposition	80.47	Current work
Oleic-acid-treated ZnO	Thermal decomposition	84.75	Current work
ZnO	Thermal decomposition	64.8	[20]
ZnO	modified polymer pyrolysis with γ-irradiation	88	[2]
ZnO	arc discharge	14 to 54	[36]
Amorphous TiO_2_	hydrolysis process	88.14	[19]
Crystalline TiO_2_	polymer pyrolysis	87	[37]

## Data Availability

All data generated or analyzed during this study are included in this published article.
